# Association of Genetic Variants in *IGF2*-Related Genes With Risk of Metabolic Syndrome in the Chinese Han Population

**DOI:** 10.3389/fendo.2021.654747

**Published:** 2021-05-20

**Authors:** Weiwei Gui, Julong Liang, Xihua Lin, Nanjing Shi, Yiyi Zhu, Bowen Tan, Hong Li

**Affiliations:** Department of Endocrinology, The Affiliated Sir Run Run Shaw Hospital, School of Medicine, Zhejiang University, Hangzhou, China

**Keywords:** metabolic syndrome, single nucleotide polymorphisms, H19, IGF2, IGF2BP2

## Abstract

**Aims:**

To explore associations between polymorphisms of *IGF2*-related genes including *H19*, *IGF2*, *IGF2BP2* and *IGF2R* and Metabolic syndrome (MetS) susceptibility in the Chinese Han population.

**Methods:**

66 subjects with MetS and 257 control subjects were collected for inclusion in a case-control study. PCR-RFLP was used to investigate polymorphisms in the *H19*, *IGF2*, *IGF2BP2* and *IGF2R* genes. Elisa was used to detect the serum *IGF2* concentrations.

**Results:**

Females carrying the GG and AG genotypes of rs680 (*IGF2)* exhibited a lower risk of MetS, compared with those harboring AA (adjusted OR = 0.388, *p* = 0.027), while GG and AG genotypes were associated with lower fasting glucose and HbA1c. In males, the Waist-to-Hip Ratio (WHR) and the level of TG were significantly higher in GG and AG genotypes than in the AA genotype of rs680 in *IGF2*. Levels of HDL-c were lower in men with GG and AG genotypes compared with those carrying the AA genotype. Serum *IGF2* concentrations did not change among different genotypes. Finally, multifactor dimensionality reduction (MDR) analysis identified interactions between four polymorphisms: rs3741279 (*H19*), rs680 (*IGF2*), rs1470579 (*IGF2BP2*) and rs629849 (*IGF2R*).

**Conclusions:**

Our study suggests that *IGF2*-related genes including *H19*, *IGF2*, *IGF2BP2* and *IGF2R* genes may play pivotal roles in the development of MetS.

## Introduction

Metabolic syndrome (MetS) is a disorder that encompasses a group of symptoms including central obesity, hypertension, hyperglycemia and dyslipidemia (1). The incidence of MetS has increased widely in recent years and always parallels the incidence of obesity and type 2 diabetes (T2DM). According to the International Diabetic Federation (IDF) diabetes atlas, the prevalence of global diabetes was 8.8% in 2015 and is predicted to increase to 10.4% by 2040. Though it’s difficult to measure the global incidence of MetS, it is estimated that around one quarter of the world population has MetS, since MetS is about three times more common than diabetes (2). The 2010–2012 China National Nutrition and Health Survey (CNNHS) showed that the overall standardized prevalence of MetS was 24.2% in China during that time-period (3).

There is growing correlational evidence that polygenic inheritance may be an important underlying mechanism in the development of MetS. Multiple genetic studies have identified numerous mutations that are related to MetS; in particular, genome-wide association studies (GWASs) have maximally revealed MetS-related genetic variants (4–6).

IGF2 is a part of the insulin-like growth factor (IGF) system which is a complex composed of two peptide hormones (*IGF-1* and *IGF-2*), two receptors (*IGF-1R* and *IGF-2R*) and six IGF binding proteins (*IGFBP1-6*) (7). The concentration of IGF2 is three times higher than that of IGF1, but our understanding of IGF2’s functions lags behind our understanding of IGF1 (8). Recent studies have identified IGF2’s role in diabetes and fatty liver (9, 10). With regard to glucose homeostasis, *IGF2BP2* is reported to be correlated with serum insulin levels and fasting blood glucose levels (11, 12). Furthermore, GWAS identified a relationship between the variants of *IGF2BP2* and risk of T2DM and its complications (13, 14). Similarly, *IGF2R* has been shown to play key roles in glucose metabolism (15, 16). Our previous work confirmed the associations between *IGF2R* and MetS (17). Long non-coding RNA H19 (*H19*) is an imprinted gene located downstream of *IGF2* that regulates the transcription and translation of *IGF2* (18, 19). *H19* is a vital regulator of glucose and lipid metabolism in T2DM and obese individuals (20, 21). In view of the pivotal role of IGF systems and *H19* in metabolic processes, it is probable that genetic variants of these genes may at least partially explain the occurrence of MetS.

In this study, we selected one or two common single nucleotide polymorphisms (SNP) of each of these genes (*H19*, *IGF2*, *IGF2BP2* and *IGF2R*) and analyzed associations between these variants and the risk of MetS in the Chinese Han population. Additionally, we estimated the role of interactions between these genes in the risk of MetS.

## Methods

### Study Population

This case-control study was approved by the ethics committee of Sir Run Run Shaw Hospital and was conducted from March to May 2010. The case participants and control participants in our study were recruited from community-based epidemiological studies of diabetes and related metabolic disorders. The study was carried out in the Caihe and Gongshu communities of Hangzhou, Zhejiang province, China. The inclusion criteria for MetS cases were in accordance with standards established by the Joint Committee for Developing Chinese Guidelines on Prevention and Treatment of Dyslipidemia in Adults (JCDCG) (22). This standard was established based on Chinese Han population’s demographic characteristics and was most suitable for our study samples. In brief, individuals with ≥3 of the following abnormalities were considered to have MetS: Central obesity [waist circumference (WC), >90 cm for men and >85 cm for women]; hypertriglyceridemia (≥1.70 mmol/l); high density lipoprotein cholesterol (HDL c; <1.04 mmol/l); elevated blood pressure (BP; ≥130/85 mmHg or current treatment for hypertension); and hyperglycemia [fasting plasma glucose (FPG) ≥6.1 mmol/l or 2 h postprandial glucose (2 h PG) ≥7.8 mmol/l]. All subjects had no previous history of cancer, cardiovascular disease, chronic inflammatory disease or renal disease. Among all the participants, 66 were diagnosed with Metabolic Syndrome (MetS) and 257 were non-MetS controls.

All participants underwent informed consent and were interviewed face-to-face by trained medical staff to collect participant demographic data, baseline lifestyle and health status data using a standardized questionnaire.

### Measurements of Clinical Traits

Participants who were not diagnosed as diabetic received a 75 g oral glucose tolerance test (OGTT), while participants who had previously been diagnosed as diabetic were administered a 100 g carbohydrate (steamed bread meal) test. Venous blood samples were obtained at 0 and 2 h after either OGTT or steamed bread meal test for subsequent biochemical tests including FPG, 2hPG, total cholesterol (TC), high density lipoprotein-cholesterol (HDL-c), low density lipoprotein-cholesterol (LDL-c), triglyceride (TG), uric acid (UA), urine albumin-to-creatinine ratio (UACR), serum creatinine (CREA), and serum urea nitrogen (BUN) using an autoanalyzer (Aeroset, Chicago, IL, USA). Blood samples for subsequent laboratory analyses were centrifuged immediately and the collected serum was stored at −80 °C. Glycosylated hemoglobin A1c (HbA1c) was measured by ion-exchange high-performance liquid chromatography (Hemoglobin Testing System; Bio-Rad, Hercules, CA, USA). Plasma insulin evels were measured using a radioimmunoassay kit (Beijing North Institute of Biological Technology, China). The homeostatic model assessment of insulin resistance (HOMA-IR) value was used to evaluate the level of insulin sensitivity and calculated as follows: fasting blood glucose (mmol/L) × fasting serum insulin (mU/L)/22.5 (23).

Physical examinations were carried out on all participants by physicians according to standard methods, including measurements of height, weight, waist, and blood pressure. Blood pressure was measured in triplicate and then averaged. Body mass index (BMI) was calculated by dividing body weight by height squared. MRI scans were performed at the level of the umbilicus between L4 and L5 with the subject in a supine position. Abdominal visceral fat area (VFA) and abdominal subcutaneous fat area (SFA) were calculated using SliceOmatic software (version 4.2).

### DNA Extraction

Peripheral blood (5 ml) was collected from all MetS patients and healthy controls. Genomic DNA was extracted using a DNA extraction kit (Tiangen Biotech; Beijing, China) by following the manufacturer’s instructions and stored at −80°C until use.

### Serum *IGF2* Concentrations Detection 

The serum *IGF2* concentrations were detected using an enzyme-linked immunosorbent (Elisa) assay kit (E-EL-H6037, Elabscience, China) according to the manufacturer’s protocol.

### SNP Selection and Genotyping

**Figure 1:**
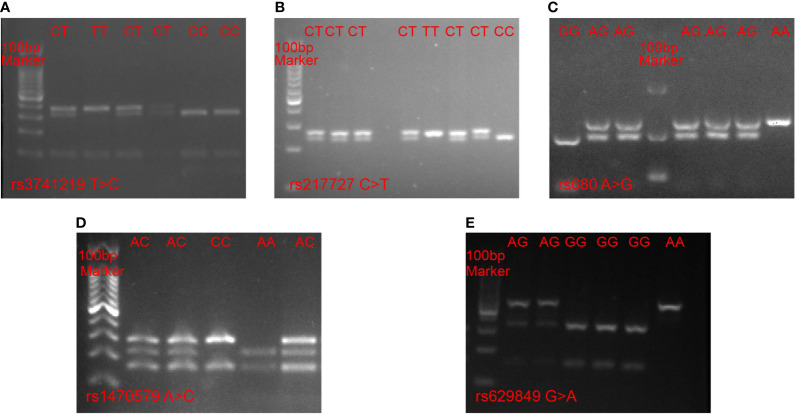
PCR-RFLP analysis of five SNPs. **(A)** rs3741219 T > C in the H19 gene, **(B)** rs217727 C > T in H19 gene, **(C)** rs680 A > G in IGF2 gene, **(D)** rs1470579 A > C in IGF2BP2 gene, **(E)** rs629849 G > A in IGF2R gene.

Several metabolism-related SNPs were chosen based on previous reports or bioinformatic analyses. The rs3741219 and rs217727 SNPs in the *H19* gene were previously reported to be closely related to type 2 diabetes mellitus (T2DM) (24), while the rs1470579 SNP in the *IGF2BP2* gene and the rs680 SNP in the *IGF2* gene have been demonstrated to play pivotal roles in the development of T2DM (25), and the rs629849 SNP in the *IGF2R* gene was reported to be associated with obesity (26). Therefore, we selected these SNPs for analysis in our study. Genotyping of the selected SNPs was performed using PCR-RFLP. The primers designed for each SNP are listed in **Supplementary Table 1**. For each amplification reaction, we used 25 µl PCR reaction mixtures consisting of 10 µg genomic DNA, 5 pmol of each primer and 1× PCR mix, and we utilized reaction setting and annealing temperatures for each SNP as previously reported (27–29). All PCR products were digested with specific restriction enzymes and the fragments were resolved by electrophoresis on a 3% agarose gel (**Figure 1**). To confirm the PCR-RFLP genotyping results, 20% of the samples were randomly selected for repeated assays, and no discrepancies were detected between the repeat analyses.

### Statistical Analyses

A Hardy–Weinberg equilibrium (HWE) was evaluated to compare the observed and expected frequencies in MetS and non-MetS control subjects by means of a chi-squared (χ^2^) test. Only SNPs that passed this test (*p >*0.05) were included in the following analyses. All continuous variables are reported as the mean ± standard deviation (SD), and variables that are not normally distributed are expressed as median value (interquartile range). Standard’s-test was used to estimate differences in the distribution of demographic characteristics between case and control subjects for continuous variables, and Fisher’s exact test was performed for categorical variables. Logistic regression was used to evaluate the association between each SNP and the risk of MetS after adjusting for potential covariates including age, smoking and drinking status. A two-sided test with type error level (α) set at 5% was used in all statistical analyses.

Furthermore, multifactor dimensionality reduction (MDR) was used to analyze SNP-SNP interactions, cross-validation consistency (CVC), the testing balanced accuracy and the sign test. Considering the influence of linkage disequilibrium (LD) among SNPs of the same gene, we selected one SNP from each high LD pair (r^2^ >0.5). Results were regarded as significant when *p <*0.05. All statistical analyses were performed in SPSS (version 26.0 for Mac; SPSS Inc, Chicago, IL, USA).

## Results

### Population Characteristics

The detailed clinical and biochemical parameters of all participants are described in **Table 1**. The mean age for MetS patients and non-MetS controls were 66.69 ± 8.71 and 62.84 ± 6.30 years, respectively. As expected, participants with MetS had more risk factors than non-MetS controls, including higher BMI, fasting glucose, 2 h plasma glucose, fasting insulin, 2 h serum insulin, HOMA-IR, HbA1c, blood pressure, triglycerides, HDL-c and levels of fat contents (*p <*0.05 for all participants). However, there were no significant differences in sex, smoking status, drinking status, LDL-c, CREA, or BUN between the two groups.

**Table 1:** Baseline Characteristics of the study samples.

Variables	MetS	Control	p
Samples, N (%)	66 (20.43)	257 (79.57)	–
Male/Female, N	34/32	100/157	0.064
Current smoker, N (%)	21(31.82)	64 (24.90)	0.255
Alcohol drinker, N (%)	35(53.03)	114 (44.36)	0.207
Age (years)	66.69 ± 8.71	62.84 ± 6.30	**<0.001**
BMI(kg/m^2^)	25.98 ± 2.71	22.94 ± 2.52	**<0.001**
WC (cm)	90.63 ± 6.23	80.65 ± 7.87	**<0.001**
WHR	0.97 ± 0.05	0.90 ± 0.06	**<0.001**
Fat% (%)	32.81 ± 7.04	28.18 ± 6.35	**<0.001**
SFA (cm^2^)	171.7 (139.15,221.10)	146.80 (113.25,191.00)	**<0.001**
VFA (cm^2^)	114.00 (89.49,155.33)	63.39 (40.54,101.95)	**<0.001**
SBP (mm Hg)	132.27 ± 14.20	120.21 ± 14.54	**<0.001**
DBP (mm Hg)	84.39 ± 8.43	79.78 ± 8.45	**<0.001**
Fasting glucose (mmol/L)	5.33 (4.89,6.21)	4.78 (4.50,5.17)	**<0.001**
2 h plasma glucose (mmol/L)	8.03 (5.58,10.26)	5.33 (4.28,6.22)	**0.002**
Fasting insulin (mIU/L)	23.22 (18.33,28.73)	17.28 (13.54,22.20)	**<0.001**
2 h insulin (mIU/L)	74.22 (41.44,133.81)	54.84 (37.14,75.61)	**<0.001**
HOMA-IR	5.60 (4.26,7.90)	3.64 (2.85,4.93)	**<0.001**
HbA1_c_ (%)	6.0 (5.6,6.4)	5.6 (5.3,5.9)	**<0.001**
TC (mmol/L)	6.06 ± 1.18	5.57 ± 1.09	**0.001**
TG (mmol/L)	2.13 (1.70,2.72)	1.23 (0.94,1.57)	**<0.001**
HDL-C (mmol/L)	1.26 ± 0.33	1.51 ± 0.36	**<0.001**
LDL-C (mmol/L)	2.52 ± 0.73	2.41 ± 0.58	0.253
CREA (μmol/L)	0.80 (0.70,0.90)	0.80 (0.70,0.90)	0.541
BUN (mmol/L)	17.00 (14.00,20.00)	16.00 (14.00,18.00)	0.095
UACR (mg/mmol)	6.00 (4.09,13.99)	4.60 (3.00,7.01)	**<0.001**

Bold indicated significant statistical difference.

MetS, Metabolic Syndrome; BMI, Body mass index; WC, Waist circumference; WHR, Waist-to-hip ratio; SFA, Subcutaneous fat area; VFA, Visceral fat area; SBP, Systolic blood pressure; DBP, Diastolic blood pressure; HOMA-IR, Homeostasis model assessment for insulin resistance; HbA1c, Glycosylated hemoglobin A1c; TC, Total cholesterol; TG, Triglyceride; HDL-c, High density lipoprotein-cholesterol; LDL-c, Low density lipoprotein-cholesterol; CREA, Serum creatinine; BUN, Serum urea nitrogen; UACR, Urine albumin-to-creatinine ratio.

### Associations of the Five SNPs With Susceptibility to MetS

Considering that *H19* and *IGF2* are imprinted genes which are regulated and expressed in a sex-specific manner, we divided our data by sex in our follow-up analyses. Logistic regression analysis was performed to evaluate the association of the five SNPs with susceptibility to MetS using both dominant and additive models after adjusting for age, smoking and drinking status. Genotype frequencies of the five SNPs of both sexes are presented for MetS patients and healthy controls in **Supplementary Table 2**. In females, frequencies of the AA, AG and GG genotypes in the rs680 SNP were 35.03, 54.78 and 10.19% in the MetS group, and 59.38, 37.50 and 3.12% in the non-MetS control group, respectively, and this difference was statistically significant (*p* = 0.036). However, there were no obvious significant differences in genotype frequencies of other SNPs including rs3741219, rs217727, rs1470579 and rs629849 between MetS and non-MetS control samples. In males, there were no statistically significant differences between the two groups in genotype frequencies of any of the five SNPs. All genotype distributions for each group were in Hardy–Weinberg equilibrium.

Results of associations between SNPs and the risk of MetS in the females are shown in **Table 2**. There were no significant associations for rs3741219, rs217727 in the *H19* gene, or for rs629849 in the *IGF2R* gene and rs1470579 in the *IGF2BP2* gene for either sex. However, there was a significant association between the rs680 SNP of the *IGF2* gene and the risk of MetS in females, though not in males (**Supplementary Table 3**). Compared to the AA genotype, females with the AG genotype exhibited a decreased risk of MetS in the additive model (crude OR = 0.404 (0.182–0.897), *p* = 0.026, adjusted OR = 0.388 (0.168–0.897), *p* = 0.027). When analyzed in a dominant model including GG and AG genotypes of rs680, a lower risk of MetS was observed compared with the homozygous variant AA genotype (crude OR = 0.369 (0.169–0.803), *p* = 0.012, adjusted OR = 0.340 (0.150–0.767), *p* = 0.01).

**Table 2:** Associations of all the SNPs of *H19, IGF2, IGF2BP2* and *IGF2R* with risk of MetS in female samples.

		MetS^a^ N (%)	Control^a^ N (%)	*p*	Crude OR (95% CI)	*p*	Adjusted^b^ OR (95% CI)
rs3741219	TT	9 (28.12)	60 (38.22)	–	1.00 (ref.)	–	1.00 (ref.)
	CT	19 (59.38)	73 (46.50)	0.211	1.564 (0.638–3.834)	0.330	1.563 (0.636–3.841)
	CC	4 (12.50)	24 (15.28)	0.871	1.044 (0.283–3.857)	0.835	1.151 (0.306–4.325)
	CC+CT	23 (71.88)	97 (61.78)	0.283	1.581 (0.686–3.644)	0.385	1.471 (0.616–3.512)
rs217727	CC	8 (25.00)	35 (22.29)	–	1.00 (ref.)	–	1.00 (ref.)
	CT	15 (46.88)	91 (57.96)	0.497	0.721 (0.281–1.851)	0.914	1.056 (0.388–2.875)
	TT	9 (28.12)	31 (19.75)	0.661	1.270 (0.437–3.696)	0.523	1.439 (0.471–4.403)
	CT+TT	24 (75.00)	122 (77.71)	0.739	0.861 (0.356–2.083)	0.729	1.179 (0.463–3.002)
rs680	AA	19 (59.38)	55 (35.03)	**–**	**1.00 (ref.)**	**–**	**1.00 (ref.)**
	AG	12 (37.50)	86 (54.78)	**0.026**	**0.404 (0.182–0.897)**	**0.027**	**0.388 (0.168–0.897)**
	GG	1 (3.12)	16 (10.19)	0.108	0.181 (0.022–1.458)	0.062	0.131 (0.015–1.109)
	AG + GG	13 (40.62)	102 (64.97)	**0.012**	**0.369 (0.169–0.803)**	**0.01**	**0.340 (0.150–0.769)**
rs1470579	AA	14 (43.75)	57 (36.31)	–	1.00 (ref.)	–	1.00 (ref.)
	AC	16 (50.00)	91 (57.96)	0.407	0.716 (0.325–1.577)	0.578	0.790 (0.345–1.809)
	CC	2 (6.25)	9 (5.73)	0.905	0.905 (0.176–4.664)	0.696	0.707 (0.125–4.016)
	AC + CC	18 (56.25)	100 (63.69)	0.429	0.733 (0.339–1.584)	0.55	0.781 (0.348–1.754)
rs629849	GG	15 (46.88)	92 (58.60)	–	1.00 (ref.)	–	1.00 (ref.)
	AG	17 (53.12)	62 (39.49)	0.183	1.682 (0.782–3.615)	0.256	1.591 (0.714–3.545)
	AA	0 (0.00)	3 (1.91)	0.999	–	0.999	–
	AG + AA	17 (53.12)	65 (41.40)	0.225	1.604 (0.748–3.442)	0.315	1.506 (0.678–3.344)

Bold indicated significant statistical difference.

a The observed genotype frequencies of SNPs among the MetS and Control were all in agreement with the Hardy–Weinberg equilibrium (*p* > 0.05 for all).

b Adjusted for age, smoking and drinking.

MetS, Metabolic Syndrome.

### Influence of rs680 on Anthropometric, Clinical Characteristics and Serum IGF-II Concentrations

We further analyzed the possible influences of polymorphism in the *IGF2* gene on anthropometric and clinical features and serum IGF-II concentrations. In females, rs680 was significantly correlated with plasma glucose level under the dominant model. Compared to the AA genotype, the GG and AG genotypes were negatively correlated with fasting glucose levels (mmol/L) (4.94 (4.61, 5.51) *vs* 4.78 (4.50, 5.17), *p* = 0.022), and HbA1c (%) (5.70 (5.40, 6.03) *vs* 5.50 (5.30, 5.80), *p* = 0.043). Furthermore, the rs680 SNP was also significantly correlated with LDL-c levels: individuals carrying either the GG or AG genotype exerted lower LDL-c level than those carrying the AA genotype (2.42 ± 0.59 *vs* 2.62 ± 0.69, *p* = 0.040). However, no other significant correlations were observed between rs680 and clinical traits in females (**Table 3**). In males, individuals with the GG or AG genotype had statistically higher WHR and TG levels and lower HDL-c level than those with the AA genotype (**Supplementary Table 4**). Serum IGF2 quantitation was detected using IGF2 Elisa kit. The results showed that individuals with AA genotype had higher serum IGF2 concentrations than those with AG and GG genotypes (206.78±68.65 ng/mL vs 179.79±56.05 ng/mL vs 174.36±39.99 ng/mL). However, the difference did not reach the statistical significance (**Supplementary Table 5**).

**Table 3:** Associations of the IGF2 rs680 clinical traits in female samples.

	AA	AG + GG	*p*
Age (years)	63.21 ± 6.87	63.38 ± 6.29	0.863
BMI (kg/m^2^)	23.15 ± 2.66	23.19 ± 2.94	0.933
WC (cm)	81.57 ± 8.64	81.67 ± 8.93	0.942
WHR	0.91 ± 0.07	0.91 ± 0.07	0.877
Fat% (%)	31.80 ± 5.94	31.92 ± 6.13	0.892
SFA (cm^2^)	186.00 (139.83,232.10)	179.60 (137.90,221.70)	0.488
VFA (cm^2^)	59.08 (35.29,84.78)	62.44 (45.81,79.70)	0.448
SBP (mm Hg)	118.94 ± 15.04	120.34 ± 15.64	0.544
DBP (mm Hg)	78.33 ± 8.33	78.94 ± 9.15	0.645
Fasting glucose (mmol/L)	4.94 (4.61,5.51)	4.78 (4.50,5.17)	**0.022**
2 h plasma glucose (mmol/L)	5.64 (4.89,7.24)	5.44 (4.56,6.44)	0.200
Fasting insulin (mIU/L)	19.74 (15.48,25.39)	18.49 (14.25,23.13)	0.216
2 h insulin (mIU/L)	62.15 (45.30,83.72)	55.14 (42.87,82.83)	0.372
HOMA-IR	4.45 (3.36,6.16)	3.98 (2.99,5.01)	0.067
HbA1_c_ (%)	5.7 (5.4,6.0)	5.5 (5.3,5.8)	**0.043**
TC (mmol/L)	5.91 ± 1.19	5.62 ± 1.11	0.081
TG (mmol/L)	1.22 (0.76,1.77)	1.23 (0.93,1.63)	0.894
HDL-C(mmol/L)	1.55 ± 0.36	1.61 ± 0.35	0.266
LDL-C(mmol/L)	2.62 ± 0.69	2.42 ± 0.59	**0.040**
CREA(μmol/L)	0.70 (0.68,0.80)	0.70 (0.70,0.70)	0.531
BUN(mmol/L)	16.00 (13.75,18.00)	16.00 (13.00,18.00)	0.993
UACR(mg/mmol)	5.68 (3.36,11.72)	5.31 (3.46,8.04)	0.282

Bold indicated significant statistical difference.

MetS, Metabolic Syndrome; BMI, Body mass index; WC, Waist circumference; WHR, Waist-to-hip ratio; SFA, Subcutaneous fat area; VFA, Visceral fat area; SBP, Systolic blood pressure; DBP, Diastolic blood pressure; HOMA-IR, Homeostasis model assessment for insulin resistance; HbA1c, Glycosylated hemoglobin A1c; TC, Total cholesterol; TG, Triglyceride; HDL-C, High density lipoprotein-cholesterol; LDL-C, Low density lipoprotein-cholesterol; CREA, Serum creatinine; BUN, Serum urea nitrogen; UACR, Urine albumin-to-creatinine ratio.

### Interaction Analyses

To further evaluate the impact of SNP-SNP interaction among all the SNPs of *H19*, *IGF2BP2*, *IGF2R* and *IGF2* on MetS risk, we performed an MDR analysis. The results obtained from the MDR analysis for two- to four-locus models are presented in **Table 4**. We found a significant three-locus model involving *H19* (rs3741219), *IGF2BP2* (rs1470579), and *IGF2* (rs680) which had the highest testing accuracy (0.6075) and exhibited good CVC (10/10), indicating a potential SNP-SNP interaction. Additionally, a two-locus model containing *IGF2BP2* (rs1470579) and *IGF2* (rs680), and a four-locus model containing *H19* (rs3741219), *IGF2BP2* (rs1470579), *IGF2* (rs680) and *IGF2R* (rs629849) also had high testing accuracy and good CVC.

**Table 4:** Best gene–gene interaction models of H19/IGF2/IGF2BP2/IGF2R pathway genes by the multifactor dimensionality reduction (MDR) in female samples.

Locus no.	Best combination	CVC	Testing accuracy	*p*
2	rs1470579 rs680	10/10	0.5949	**0.0010**
3	rs3741219 rs1470579 rs680	10/10	0.6075	**0.0107**
4	rs3741219 rs1470579 rs629849 rs680	10/10	0.5374	**0.0010**

Bold indicated significant statistical difference.

Gene–environment interactions between SNPs and clinical characteristics of MetS were analyzed by MDR. The results obtained from the MDR analysis for one- to four-locus models are presented in **Supplementary Tables 6**–**9**. We found that blood glucose, blood pressure, WC and triglycerides were independent risk factors for MetS. Furthermore, we found a significant three-locus model involving *H19* (rs3741219), *IGF2* (rs680) and blood glucose which had the highest testing accuracy (0.7853) and exhibited good CVC (10/10). The three-locus model involving *IGF2BP2* (rs1470579), *IGF2* (rs680) and blood pressure had the highest testing accuracy (0.7574) and good CVC (10/10). As for WC, two-locus model involving *H19* (rs3741219) and WC had high testing accuracy and good CVC. Finally, four-locus model involving *H19* (rs3741219), *IGF2BP2* (rs1470579), *IGF2* (rs680) and triglycerides exhibited good CVC which indicated the SNPs-triglycerides interactions.

## Discussion

Metabolic syndrome (MetS) is regarded as a polygenic metabolic disorder accompanied by abnormal lipid and glucose metabolism (30). Few diseases are determined purely by a single genetic or environmental factor, especially chronic diseases. For this reason, if we want to understand disease susceptibility, it is necessary to study gene–gene or gene–environment interactions. In the case of MetS, however, most of the previous literature has focused on association studies exploring the contribution of single polymorphisms to the development of MetS. It’s more plausible to study the association between multiple genes in the same signaling pathway or the same system and the risk of MetS. In this study, we thus explored systematically the association between several *IGF2*-related genes and MetS susceptibility in the Chinese Han population for the first time. Our findings will help to detect the roles of IGF2-related genes including IGF2, IGF2BP2, IGF2R and H19 in the etiology of MetS. To increase the detection rate, we selected representative SNPs which have previously been reported to be related to the development of metabolic disease, such T2DM and obesity. In the present study, we identified the AG and GG genotypes of rs680 in the *IGF2* gene as candidate resistant factors to the development of MetS in females. Although no effects were detected for SNPs in the *H19*, *IGF2BP2* and *IGF2R* genes on the occurrence of MetS, interactions between *H19*, *IGF2*, *IGF2BP2* and *IGF2R* genes on MetS were confirmed by MDR analyses.

There is growing evidence indicating that the IGF system plays an important role in glucose and lipid metabolism (31, 32). Furthermore, because concentrations of IGF vary with age, sex and pregnancy status (33), we analyzed our data according to gender. Interestingly, we found that, for women, but not for men, the GG and AG genotypes of rs680 in *IGF2* were protective factors for MetS, and were negatively related with fasting glucose and HbA1c. However, in males, the GG and AG genotypes of rs680 in *IGF2* were positively correlated with WHR and TG while negatively related with levels of HDL-c. Though several studies have previously revealed an association between *IGF2* (rs680) and obesity, BMI or plasma glucose levels (34–36), these results were previously controversial, and the association between rs680 and the risk of MetS was still unclear. The controversy between the gender in our study might be due to the fact that *IGF2* is an imprinted gene whose transcription is achieved by methylation of the differentially methylated region (DMR) on the maternal allele (8). Heijmans et al. (37) reported that the rs680 SNP in *IGF2* is strongly related to methylation of multiple CpG sites within the *IGF2* DMR, while another study reported that the variant allele is associated with higher serum *IGF2* concentrations than the wide type allele in middle-aged men (34). *IGF2* is regarded as an important regulator of adipocyte physiology and its high level is closely associated with being overweight (35, 38), which might partially explain our results. As for females, Gatford et al. (39) reported that pregnant women with variant allele of rs680 in *IGF2* exhibited lower circulating *IGF1* concentration than those with the wide type allele. However, this effect was not observed in non-pregnant women (39). *IGF1* has been reported to play an important role in maintaining normal glucose homeostasis (40). Thus, we postulate that *IGF2* polymorphism probably influences the *IGF1* or *IGF2* levels which ultimately regulate glucose metabolism. To confirm our speculation, we detected serum IGF2 levels using Elisa kit and we found a decreasing tendency in serum *IGF2* concentrations of individuals with GG genotype, though the data did not have statistical significance. SD O'Dell et al. (34) reported that serum *IGF2* level in males with GG was lower than those with AA genotype. But Rashad et al. (27) found no differences in serum *IGF2* levels between GG and AA genotypes. The contradiction among these studies may be attribute to the different ethnicity. However, more experimental evidence is needed to confirm this finding.

Previous studies have reported relationships between *H19* (rs217727), *IGF2BP2* (rs1470579) and T2DM susceptibility (24, 25, 41). Yang et al. revealed that the minor allele “A” in rs629849 is associated with obesity in the Korean population (26). One puzzling aspect of our findings in this study is that we failed to find any statistically significant associations between these three polymorphisms and MetS susceptibility. This contradiction might be due to heterogeneity in samples and environmental factors between the studies, and our limited sample size.

*IGF2BP2* was reported to regulate *IGF2* mRNA translation *via* binding to *IGF2* L3 5’ UTR as an essential IRES trans-acting factor (ITAF) (42, 43). *H19* harbored four binding sites with *IGF2BP2* mRNA (19) and shared the same loci with *IGF2* which indicated the interactions among these three genes. MDR is a newly established approach mainly used to detect the combined effects of multiple factors in disease susceptibility. Traditional statistical methods like logistic regression analysis encounter dimension problems in studying gene–gene interactions which could be solved by the use of MDR. In our study, we showed through MDR analysis that there is an interplay among *H19* (rs3741219), *IGF2BP2* (rs1470579), and *IGF2* (rs680), indicative of the interactive role of these polymorphisms in MetS occurrence, which is consistent with the abovementioned reports. In view of the significant associations between rs680 in *IGF2* and risk of MetS in single locus analysis, we speculate that the *IGF2* gene plays a major part in the interaction. Furthermore, we found the interactions between each SNPs and clinical characteristics of MetS.

There are some limitations in the present study. Firstly, our sample size was relatively small. Despite this, significant differences between groups were still detected and we believe that a larger sample size would further confirm our results. Secondly, the study was based on observational and as such was particularly vulnerable to potential biases such as information or selection bias. Thirdly, we did not measure the gene expression activities of each polymorphism. Finally, though we used MDR as a bioinformatics method for identifying gene–gene interactions, it wasn’t possible to quantitatively assess the disease risk of the genotype combinations. A new weighted risk score-based multifactor dimensionality reduction (WRSMDR) analysis seems like a good and feasible option for the assessment of disease risk in future studies (44).

## Conclusion

In conclusion, our work systematically studied the association between polymorphisms in four genes (*H19*, *IGF2*, *IGF2BP2* and *IGF2R*) with risk of MetS in the Chinese Han population. The *IGF2* variant rs680 was negatively correlated with fasting glucose and HbA1c, and might be a promising protective variant for the risk of MetS in females. In males, we failed to find the association between the rs680 SNP in *IGF2* and the risk of MetS. While GG genotype of *IGF2* rs680 was positively correlated with WHR and TG, and negatively correlated with HDL-c. Moreover, this study confirmed that interactions occur among the *H19*, *IGF2*, *IGF2BP2* and *IGF2R* genes, and supported a previously-reported correlation between this system and the risk of MetS. Our conclusions should be further evaluated with a larger size sample.

## Data Availability Statement

The original contributions presented in the study are included in the article/**Supplementary Material**. Further inquiries can be directed to the corresponding author.

## Author Contributions

WG: Conceptualization, methodology, investigation, and writing—original draft writing. JL: Methodology and investigation. XL: Conceptualization, methodology, and supervision. NS: Investigation and validation. YZ: Investigation and validation. BT: Conceptualization and methodology. HL: Conceptualization, supervision, writing—review and editing, and funding acquisition. All authors contributed to the article and approved the submitted version.

## Funding

This work was supported by grants from the National Natural Science Foundation of China (81873653).

## Conflict of Interest

The authors declare that the research was conducted in the absence of any commercial or financial relationships that could be construed as a potential conflict of interest.

## Supplementary Material

Supplementary Table 1 PCR Primer sequences in gene polymorphisms.

Supplementary Table 2 The Genotypic Frequency of Polymorphisms Between MetS and non-MetS Groups.

Supplementary Table 3 Associations of all the SNPs of *H19, IGF2, IGF2BP2* and *IGF2R* with risk of MetS in male samples.

Supplementary Table 4 Associations of the IGF2 rs680 clinical traits in male samples.

Supplementary Table 5 Associations of the IGF2 rs680 with serum *IGF2* concentrations in the whole samples.

Supplementary Table 6 Best gene–gene interaction models of H19/IGF2/IGF2BP2/IGF2R pathway genes and blood glucose by the multifactor dimensionality reduction (MDR) in female samples.

Supplementary Table 7 Best gene–gene interaction models of H19/IGF2/IGF2BP2/IGF2R pathway genes and blood pressure by the multifactor dimensionality reduction (MDR) in female samples.

Supplementary Table 8 Best gene–gene interaction models of H19/IGF2/IGF2BP2/IGF2R pathway genes and WC by the multifactor dimensionality reduction (MDR) in female samples.

Supplementary Table 9 Best gene–gene interaction models of H19/IGF2/IGF2BP2/IGF2R pathway genes and triglycerides by the multifactor dimensionality reduction (MDR) in female samples.
