# Supplementary material for: Association of Genetic Variants in IGF2-Related Genes With Risk of Metabolic Syndrome in the Chinese Han Population
Source: Front Endocrinol (Lausanne). 2021 May 20;12:654747. doi: 10.3389/fendo.2021.654747 (PMC8173176; doi:10.3389/fendo.2021.654747)
Supplement: Supplementary Table 1 — PCR Primer sequences in gene polymorphisms. [file Table_1.docx]

**Supplementary Table 1** PCR Primer sequences in gene polymorphisms

| **Polymorphisms** | **Primer sequences** |
| --- | --- |
| *H19*-rs37412191 T>C | Forward: 5'-CCCCCTGCGGCGGACGGTTGA-3' |
|  | Reverse: 5'-GGCGTAATGGAATGCTTGAA-3' |
| *H19*-rs217727 C>T | Forward: 5'-ACTCAGGAATCGGCTCTGGAAGGTG-3' |
|  | Reverse: 5'-GATGTGGTGGCTGGTGGTCAACGGT-3' |
| *IGF2*-rs680 A>G | Forward: 5'-CTTGGACTTTGAGTCAAATTGG-3' |
|  | Reverse: 5'-GGTCGTGCCAATTACATTTCA-3' |
| *IGF2BP2*-rs1470579 A>C | Forward:5'- CAGGGGTAGATGATGTAAGTGGT-3' |
|  | Reverse:5'- ACCTAATTTGATTTTGAGTTTCC-3' |
| *IGF2R*-rs629849 G>A | Forward:5'- AACAATGGTTAAAGCCGGATTG-3' |
|  | Reverse:5'- GGCCCGGGTGCAGCCAGGCACTG-3' |

**Supplementary Table 2. The Genotypic Frequency of Polymorphisms Between MetS and non-MetS Groups**

| **Polymorphisms** | **Genotype** | **Male** | | | **Female** | | |
| --- | --- | --- | --- | --- | --- | --- | --- |
|  |  | **MetS N %** | **non-MetS N %** | ***p* value** | **MetS N %** | **non-MetS N %** | ***p* value** |
| *H19*-rs3741219 T>C | TT | 16(47.06) | 50(50.00) | 0.054 | 9(28.12) | 60(38.22) | 0.465 |
|  | CT | 12(35.29) | 39(39.00) |  | 19(59.38) | 73(46.50) |  |
|  | CC | 6(17.65) | 11(11.00) |  | 4(12.50) | 24(15.28) |  |
| *H19*-rs217727 C>T | CC | 3(8.83) | 20(20.00) | 0.242 | 8(25.00) | 35(22.29) | 0.438 |
|  | CT | 21(61.76) | 60(60.00) |  | 15(46.88) | 91(57.96) |  |
|  | TT | 10(29.42) | 20(20.00) |  | 9(28.12) | 31(19.75) |  |
| *IGF2*-rs680 A>G | AA | 10(29.41) | 45(45.00) | 0.146 | 19(59.38) | 55(35.03) | **0.036** |
|  | AG | 18(52.94) | 47(47.00) |  | 12(37.50) | 86(54.78) |  |
|  | GG | 6(17.65) | 8(8.00) |  | 1(3.12) | 16(10.19) |  |
| *IGF2BP2*-rs1470579 A>C | AA | 15(44.12) | 43(43.00) | 0.682 | 14(43.75) | 57(36.31) | 0.653 |
|  | AC | 17(50.00) | 54(54.00) |  | 16(50.00) | 91(57.96) |  |
|  | CC | 2(5.88) | 3(3.00) |  | 2(6.25) | 9(5.73) |  |
| *IGF2R*-rs629849 G>A | GG | 19(55.88) | 55(55.00) | 0.317 | 15(46.88) | 92(58.60) | 0.324 |
|  | AG | 12(35.29) | 42(42.00) |  | 17(53.12) | 62(39.49) |  |
|  | AA | 3(8.83) | 3(3.00) |  | 0(0) | 3(1.91) |  |

**Supplementary Table 3. Associations of all the SNPs of *H19, IGF2, IGF2BP2* and *IGF2R* with risk of MetS in male samples**

|  |  | **MetS N (%)** | | **non-MetS^a^ N(%)** | ***p*** | **Crude OR (95% CI)** | ***p*** | **Adjusted OR (95% CI)** |
| --- | --- | --- | --- | --- | --- | --- | --- | --- |
| rs3741219 | TT | | 16(47.06) | 50(50.00) | - | 1.00(ref.) | - | 1.00(ref.) |
|  | CT | | 12(35.29) | 39(39.00) | 0.929 | 0.962(0.408-2.267) | 0.762 | 0.873(0.362-2.104) |
|  | CC | | 6(17.65) | 11(11.00) | 0.36 | 1.705(0.544-5.346) | 0.539 | 1.446(0.446-4.691) |
|  | CC+CT | | 18(52.94) | 50(50.00) | 0.767 | 1.125(0.516-2.452) | 0.993 | 1.003(0.451-2.231) |
| rs217727 | CC | | 3(8.83) | 20(20.00) | - | 1.00(ref.) | - | 1.00(ref.) |
|  | CT | | 21(61.76) | 60(60.00) | 0.205 | 2.333(0.629-8.659) | 0.169 | 2.548(0.672-9.662) |
|  | TT | | 10(29.42) | 20(20.00) | 0.099 | 3.333(0.797-13.948) | 0.08 | 3.809(0.850-17.067) |
|  | CT+TT | | 31(91.18) | 80(80.00) | 0.147 | 2.583(0.717-9.313) | 0.122 | 2.804(0.758-10.367) |
| rs680 | AA | | 10(29.41) | 45(45.00) | - | 1.00(ref.) | - | 1.00(ref.) |
|  | AG | | 18(52.94) | 47(47.00) | 0.222 | 1.723(0.719-4.132) | 0.177 | 1.925(0.745-4.973) |
|  | GG | | 6(17.65) | 8(8.00) | 0.059 | 3.375(0.956-11.909) | 0.054 | 3.614(0.978-13.350) |
|  | AG+GG | | 24(70.59) | 55(55.00) | 0.114 | 1.964(0.851-4.532) | 0.089 | 2.199(0.886-5.458) |
| rs1470579 | AA | | 15(44.12) | 43(43.00) | - | 1.00(ref.) | - | 1.00(ref.) |
|  | AC | | 17(50.00) | 54(54.00) | 0.802 | 0.902(0.405-2.012) | 0.6 | 0.801(0.349-1.838) |
|  | CC | | 2(5.88) | 3(3.00) | 0.5 | 1.911(0.291-12.565) | 0.643 | 1.592(0.223-11.357) |
|  | AC+CC | | 19(55.88) | 57(57.00) | 0.91 | 0.956(0.436-2.093) | 0.675 | 0.840(0.371-1.898) |
| rs629849 | GG | | 19(55.88) | 55(55.00) | - | 1.00(ref.) | - | 1.00(ref.) |
|  | AG | | 12(35.29) | 42(42.00) | 0.653 | 0.827(0.362-1.891) | 0.893 | 0.943(0.403-2.208) |
|  | AA | | 3(8.83) | 3(3.00) | 0.216 | 2.895(0.538-15.581) | 0.128 | 3.914(0.674-22.718) |
|  | AG+AA | | 15(44.12) | 45(45.00) | 0.929 | 0.965(0.441-2.112) | 0.808 | 1.106(0.493-2.482) |

Bold indicated significant correlation.

^a^: The observed genotype frequencies of SNPs among the non-MetS were all in agreement with the Hardy–Weinberg equilibrium (*P* > 0.05 for all).

**Supplementary Table 4. Associations of the *IGF2* rs680 with clinical traits in male samples**

|  | **AA** | | **AG+GG** | ***p*** |
| --- | --- | --- | --- | --- |
| Age (years) | 64.33±6.07 | 63.88±8.67 | | 0.742 |
| BMI(kg/m^2^) | 24.06±2.81 | 24.14±2.77 | | 0.871 |
| WC (cm) | 82.59±8.47 | 85.29±7.56 | | 0.056 |
| WHR | 0.91±0.06 | 0.94±0.05 | | 0.009 |
| Fat% (%) | 24.45±5.31 | 25.78±5.94 | | 0.183 |
| SFA (cm^2^) | 116.90(101.90,165.20) | 124.90(106.20,146.20) | | 0.912 |
| VFA (cm^2^) | 102.00(53.72,127.20) | 111.80(73.59,132.30) | | 0.082 |
| SBP (mm Hg) | 126.50±14.89 | 126.91±13.71 | | 0.872 |
| DBP (mm Hg) | 82.90±8.28 | 84.03±6.98 | | 0.395 |
| Fasting glucose (mmol/L) | 5.00(4.50,5.56) | 4.89(4.56,5.39) | | 0.905 |
| 2h plasma glucose (mmol/L) | 5.67(4.00,8.61) | 5.11(4.11,7.78) | | 0.731 |
| Fasting insulin (mIU/L) | 17.57(13.11,23.36) | 18.10(13.24,23.44) | | 0.957 |
| 2h insulin (mIU/L) | 52.92(25.30,87.75) | 52.99(35.87,92.51) | | 0.513 |
| HOMA-IR | 4.05(2.62,5.67) | 3.72(2.88,5.51) | | 0.923 |
| HbA1_c_ (%) | 5.6(5.4,5.9) | 5.7(5.4,6.1) | | 0.474 |
| TC (mmol/L) | 5.55±0.94 | | 5.59±1.17 | 0.859 |
| TG (mmol/L) | 1.33(1.09,1.92) | | 1.73(1.26,2.40) | **0.042** |
| HDL-C(mmol/L) | 1.34±0.33 | | 1.23±0.25 | **0.024** |
| LDL-C(mmol/L) | 2.35±0.53 | | 2.32±0.61 | 0.754 |
| CREA(μmol/L) | 0.90(0.90,1.00) | | 0.90(0.80,1.00) | 0.231 |
| BUN(mmol/L) | 18.00(15.00,21.00) | | 16.00(14.00,18.00) | 0.069 |
| UACR(mg/mmol) | 4.33(2.72,6.24) | | 4.10(2.71,5.76) | 0.74 |

Bold indicated significant correlation.

Notes: MetS: Metabolic Syndrome; BMI: Body mass index; WC: Waist circumference; WHR: Waist-to-hip ratio; SFA: Subcutaneous fat area; VFA: Visceral fat area; SBP: Systolic blood pressure; DBP: Diastolic blood pressure; HOMA-IR: Homeostasis model assessment for insulin resistance; HbA1c: Glycosylated hemoglobin A1c; TC: Total cholesterol; TG: Triglyceride; HDL-c: High density lipoprotein-cholesterol; LDL-c: Low density lipoprotein-cholesterol; CREA: Serum creatinine; BUN: Serum urea nitrogen; UACR: Urine albumin-to-creatinine ratio.

**Supplementary Table 5. Associations of the *IGF2* rs680 with serum IGF2 concentrations in the whole samples**

| **SNP** | **Genotypes** | **N** | **IGF2（ng/mL）** | ***p*** |
| --- | --- | --- | --- | --- |
| *IGF2* rs680 | AA | 27 | 206.78±68.65 |  |
|  | AG | 48 | 179.79±56.05 | 0.128 |
|  | GG | 10 | 174.36±39.99 |  |

**Supplementary Table 6. Best gene–gene interaction models of *H19*/*IGF2*/*IGF2BP2*/*IGF2R* pathway genes and blood glucose by the multifactor dimensionality reduction (MDR) in female samples**

| **Locus no.** | **Best combination** | **CVC** | **Testing accuracy** | ***p*** |
| --- | --- | --- | --- | --- |
| 1 | blood glucose | 10/10 | 0.7469 | **0.0010** |
| 2 | rs629849 blood glucose | 8/10 | 0.7302 | **0.0010** |
| 3 | rs3741219 rs680 blood glucose | 10/10 | 0.7853 | **0.0010** |
| 4 | rs3741219 rs680 rs1470579 blood glucose | 7/10 | 0.6119 | **0.0010** |

Bold indicated significant statistical difference.

**Supplementary Table 7. Best gene–gene interaction models of *H19*/*IGF2*/*IGF2BP2*/*IGF2R* pathway genes and blood pressure by the multifactor dimensionality reduction (MDR) in female samples**

| **Locus no.** | **Best combination** | **CVC** | **Testing accuracy** | ***p*** |
| --- | --- | --- | --- | --- |
| 1 | blood pressure | 10/10 | 0.7477 | **0.0010** |
| 2 | rs3741219 blood pressure | 7/10 | 0.7217 | **0.0010** |
| 3 | rs1470579 rs680 blood pressure | 10/10 | 0.7574 | **0.0010** |
| 4 | rs3741219 rs680 rs1470579 blood pressure | 9/10 | 0.6612 | **0.0010** |

Bold indicated significant statistical difference.

**Supplementary Table 8. Best gene–gene interaction models of *H19*/*IGF2*/*IGF2BP2*/*IGF2R* pathway genes and WC by the multifactor dimensionality reduction (MDR) in female samples**

| **Locus no.** | **Best combination** | **CVC** | **Testing accuracy** | ***p*** |
| --- | --- | --- | --- | --- |
| 1 | WC | 10/10 | 0.8527 | **0.0010** |
| 2 | rs3741219 WC | 8/10 | 0.8235 | **0.0010** |
| 3 | rs1470579 rs680 WC | 8/10 | 0.8218 | **0.0010** |
| 4 | rs3741219 rs629849 rs1470579 WC | 5/10 | 0.7470 | **0.0010** |

Bold indicated significant statistical difference.

Note: WC: Waist circumference.

**Supplementary Table 9. Best gene–gene interaction models of *H19*/*IGF2*/*IGF2BP2*/*IGF2R* pathway genes and triglycerides by the multifactor dimensionality reduction (MDR) in female samples**

| **Locus no.** | **Best combination** | **CVC** | **Testing accuracy** | ***p*** |
| --- | --- | --- | --- | --- |
| 1 | triglycerides | 10/10 | 0.8527 | **0.0010** |
| 2 | rs629849 triglycerides | 7/10 | 0.8235 | **0.0010** |
| 3 | rs3741219 rs680 triglycerides | 6/10 | 0.8218 | **0.0010** |
| 4 | rs3741219 rs680 rs1470579 triglycerides | 10/10 | 0.7470 | **0.0010** |

Bold indicated significant statistical difference.
